# Social Background Effects on Educational Outcomes—New Insights from Modern Genetic Science

**DOI:** 10.1007/s11577-024-00970-2

**Published:** 2024-09-13

**Authors:** Tina Baier, Torkild Hovde Lyngstad

**Affiliations:** 1 WZB—Berlin Social Science Center, Reichpietschufer 50, 10785 Berlin, Germany; 2 Einstein Center Population Diversity (ECPD), Berlin, Germany; 3 Department of Sociology and Human Geography, University of Oslo, Postboks 1096 Blindern, 0317 Oslo, Norway

**Keywords:** Social background effects, Education, Indirect genetic effects, Genetic nurture, Molecular genetics, Effekte sozialer Herkunft, Bildung, Indirekte genetische Effekte, Genetic Nurture, Molekulargenetik

## Abstract

Sociological theory and empirical research have found that parents’ socioeconomic status and related resources affect their children’s educational outcomes. Findings from behavior genetics reveal genetic underpinnings of the intergenerational transmission of education, thus altering previous conclusions about purely environmental transmission mechanisms. In recent years, studies in molecular genetics have led to new insights. Genomic data, polygenic scores, and other facets of sociogenomics are increasingly used to advance research in social stratification. Notably, the 2018 discovery of “genetic nurture” suggested that parents’ genes influence children above and beyond the genes they directly transmitted to their children. Such indirect genetic effects can be interpreted as consequences of parental behavior, which is itself influenced by the parents’ genetics and is essential for their children’s environment. Indirect genetic effects fit hand in glove with the sociological literature because they represent environmental transmission mechanisms. For instance, parenting behaviors, which are partly influenced by parents’ genes, shape children’s home environments and possibly their later educational outcomes. However, current findings based on more sophisticated research designs demonstrate that “genetic nurture” effects are actually much smaller than initially assumed and hence call for a reevaluation of common narratives found in the social stratification literature. In this paper, we review recent developments and ongoing research integrating molecular genetics to study educational outcomes, and we discuss their implications for sociological stratification research.

## Abstract (de)

Soziologische Theorien und empirische Forschung haben gezeigt, dass der sozioökonomische Status der Eltern und die damit verbundenen Ressourcen die Bildungschancen ihrer Kinder beeinflussen. Forschungsergebnisse aus der Verhaltensgenetik belegen, dass genetische Faktoren der intergenerationalen Transmission von Bildung zugrunde liegen, und ändern damit bisherige Schlussfolgerungen über rein umweltbedingte Transmissionsmechanismen. In den letzten Jahren haben Studien in der Molekulargenetik zu neuen Erkenntnissen geführt. Genetisch sensitive Daten, „polygenic scores“ und weitere Facetten aus dem Bereich „sociogenomics“ werden zunehmend eingesetzt, um Forschungen zur sozialen Stratifizierung weiterzuentwickeln. Insbesondere die Entdeckung von „Genetic Nurture“ im Jahr 2018 legte nahe, dass nicht nur genetische Faktoren, die direkt vererbt werden, einen Einfluss auf die Kinder haben, sondern auch genetische Faktoren, die nicht vererbt werden. Solche indirekten genetischen Effekte können als Konsequenzen des Verhaltens der Eltern interpretiert werden. Elterliche Verhaltensweisen werden von Genen beeinflusst und wirken sich auf die Umwelt ihrer Kinder aus. Indirekte genetische Effekte fügen sich gut in die soziologische Literatur zur Ungleichheitsforschung ein, da sie soziale Übertragungsmechanismen darstellen. Zum Beispiel prägen elterliche Verhaltensweisen, die teilweise von den Genen der Eltern beeinflusst werden, die häusliche Umgebung der Kinder und möglicherweise auch ihre späteren Bildungsergebnisse. Jedoch zeigen aktuelle Forschungsergebnisse, die auf anspruchsvolleren Forschungsdesigns basieren, dass die Effekte von „Genetic Nurture“ tatsächlich kleiner sind als zunächst angenommen und fordern daher eine Neubewertung der gängigen Erklärungsansätze in der Stratifizierungsliteratur. In diesem Beitrag berichten wir über die aktuellen Forschungsentwicklungen und laufenden Studien, die molekulargenetische Ansätze verfolgen, um verschiedene Bildungsoutcomes zu untersuchen, und diskutieren die Implikationen dieser Befunde für die soziologische Ungleichheitsforschung.

## Introduction

How parents affect their children’s educational outcomes is one of the core questions in social stratification research (Breen and Jonsson 2005; Lareau 2011; Bukodi and Goldthorpe 2022; Goldthorpe 1996; Shavit and Blossfeld 1993; Shavit et al. 2007). Theoretical accounts of how parents’ behaviors, characteristics, and resources matter for educational careers include, but are not limited to, help with homework, (bedside) reading, financial help, psychological support, after-school activities, the transmission of cultural norms and institutional knowledge, and so on (Bukodi and Goldthorpe 2022; Lareau 2011; Haller and Portes 1973; Pfeffer and Hertel 2015; Jæger 2009). There is a myriad of channels through which parents may affect their children’s outcomes. Yet most empirical contributions do not specify the underlying transmission mechanisms. Instead, social stratification scholars usually use different indicators for parents’ social standing to proxy the various transmission mechanisms. Numerous studies find that parents’ socioeconomic status measures, e.g., educational attainments, occupation, or income, are correlated with a wide range of children’s outcomes.

From a different vantage point, behavior geneticists consistently show that almost all of the well-researched stratification outcomes are at least partly heritable, i.e., significantly influenced by someone’s genes (Polderman et al. 2015). The mere existence of genetic effects on any socioeconomic status–related outcome implies that we cannot be certain whether status-related resources matter for children’s outcomes, the genes that children inherit from their parents, or even a combination of both. Thus, any empirical study on the importance of parental resources may be genetically confounded due to genetic transmission.

Over the last decade, a scholarship within the social sciences has emerged that acknowledges the role of genetic transmission and makes use of recent methodological advances rooted in molecular genetics to study outcomes related to social stratification. This line of research has predominantly used polygenic indexes (PGIs), which are based on large-scale genome-wide association studies (GWAS). They function as indicators of an individual’s genetic liability for a phenotype (an individual-level characteristic) and are constructed by summarizing genetic effects discovered in GWAS conducted for that phenotype. One clear advantage of PGIs is that they seemingly provide an easy and intuitive way of accounting for direct genetic transmission. Overall, this research demonstrates that the impact of social background indicators, such as parental education, indeed diminishes once genetic transmission is accounted for, but it is nevertheless substantial (Conley et al. 2015; Domingue et al. 2015; Liu 2018; Isungset et al. 2022).

However, these results have been questioned. Specifically, it remains unclear how much of the genetic variance is actually explained by a PGI. Per design, PGIs only carry a fraction of the genetic variants that are important for the outcome under study. Since the genetic variance captured by PGIs is small, associations between PGIs and individual outcomes may be underestimated (Pingault et al. 2022). In addition, GWAS estimates are noisy, and so are PGIs, leading to their lower efficacy as control variables due to the signal being drowned out by measurement error (attenuation bias). Finally, a PGI not only captures direct genetic influences but may also capture environmental influences and population structure. For instance, with respect to environmental influences, educational PGIs may not solely be linked to educational outcomes but are likely to also be correlated with certain parental characteristics and behaviors and/or the institutional characteristics of educational systems (Lee et al. 2018).

Delving deeper into the role of genetics in the intergenerational transmission processes, scholars have just begun to move beyond the study of direct genetic effects and analyze indirect genetic effects. Specifically, scholars have introduced a novel approach in which the simultaneous consideration of genomes of trios—mother, father, and their child—makes it possible to study not only direct effects of ego’s genes on ego’s outcomes but also indirect genetic effects (Kong et al. 2018; Bates et al. 2018). These indirect effects are effects of the parents’ genes above and beyond the children’s genes. The behavioral genetic literature refers to these as “genetic nurture.” They are “nurture” because they are assumed to be the result of the many nurturing behaviors of parents, and they are “genetic” because they flow causally from parents’ genetics. In other words, genetic nurture refers to parents’ genes shaping parents’ behaviors in ways that in turn *become *nurturing environments for their children. Genetic nurture resonates with classical social stratification research on the effects of home environments and parents’ behaviors directed toward their children.

The international meta-analysis by Wang et al. (2021) included a variety of educational outcomes and found the average estimate of indirect genetic effects to be approximately half the magnitude of the direct genetic effect. Studies have reached similar conclusions for educational achievement, i.e., test scores, using Norwegian data (Isungset et al. 2022). Thus, recent studies have found robust evidence for indirect genetic effects, although the impact of these effects is comparatively small. What is more, recent developments suggest that indirect genetic effects cannot easily be attributed to mechanisms operating within the nuclear family (e.g., parents’ behaviors; Nivard et al. 2024). Instead, indirect effects may reflect other types of social mechanisms operating outside the proximate family context and encompass multigenerational stratification processes, including patterns in mating choices. Even though the underlying mechanisms are the subject of current research, there is increasing evidence that clearly challenges conventional sociological perspectives on the origins of intergenerational correlations in educational outcomes.

In this paper, we discuss what the current wave of molecular genetic studies of education have to say about social background effects. Specifically, we focus on indirect genetic effects that currently receive a lot of attention in the literature and discuss what their presence or absence may mean for sociologists’ thinking about social background influences.

## A Primer for Modern Genetic Approaches Relevant for Social Stratification Research

For a long time, researchers had to rely on family designs to study the importance of genes. In conventional family studies, genes are not directly measured but are inferred through assumptions about their genetic relatedness and the amount of shared upbringing (Plomin et al. 2008). The most well-known design is the classical twin design (CTD), which includes monozygotic (MZ) and dizygotic (DZ) twins. In fact, most of what we know about the relative importance of genes comes from twin studies. The underlying idea is straightforward: DZ twins share, on average, 50% of their genes; MZ twins are genetically identical (at conception); and both types of twins grow up under the same (family) circumstances because there are no age differences between them. Hence, if MZs are more similar in the outcome of interest compared to DZs, it can be concluded—under certain assumptions—that genetic influences are important for explaining variation in that particular trait.

Synthesizing results based on twin designs has led to the formulation of the “[t]hree laws of behavioral genetics” (Turkheimer 2000). First, all human behavioral traits are heritable. Second, the effect of shared (family) environments is smaller than the effect of genetics. And third, a sizable portion of the variation in behavioral traits can be explained by neither individual genetics nor shared (family) environments alone, and is attributed to nonshared environmental influences (Plomin and Daniels 1987; Turkheimer and Waldron 2000). Note that the term “law” does not imply any genetic determinism, as it is widely acknowledged among researchers that the impact of genetic dispositions plays out in environments and also is shaped by environmental influences and social–institutional contexts. Instead, the three laws accommodate the empirical regularity that genetic influences matter in some way or the other for individual outcomes. The notion that genes matter is important for stratification researchers because it opens avenues for research on the intergenerational transmission of inequality, specifically, the question of whether and how these genetic effects vary by features of the social context, such as the socioeconomic status of the parents or institutional arrangements. Answers to these questions can help us uncover the mechanisms through which social inequalities are reproduced and manifested.

For several decades, stratification scholars have made increasing use of the CTD to study to what extent individual differences in stratification-related outcomes can be explained by environmental and genetic transmission mechanisms. Overall, findings confirm the comparatively weak role of shared family background influences (with the notable exception of education; see Freese and Jao 2015) and the stronger role of genetics. Yet the relative importance of these factors seems to some extent to be moderated by social contexts (Branigan et al. 2013; Baier and Lang 2019; Nielsen 2006; Erola et al. 2021).

Although the CTD and its extensions (extended twin family designs; see Keller et al. 2010) can provide valuable insights on the importance of genetics and environments across the life course and social contexts, twin studies come with rather strong assumptions (Plomin et al. 2008). The equal environment assumption (EEA) states that environmental influences relevant for the outcome under study are shared to the same extent by MZ and DZ twins, or in other words, that the degree of shared environmental variation should not differ by zygosity. This allows researchers to attribute differences in similarity between MZ and DZ twins primarily to genetic factors. Thus, it is assumed that the shared environmental influences—those environmental factors that are common to both twins in a pair—are similarly experienced by both MZ and DZ twins, thereby not differing significantly by zygosity. Numerous studies support the validity of the EEA (e.g., Conley et al. 2013; Mönkediek 2021; Felson 2014). Thus, this assumption does not impose a major threat to studies based on twin data. In addition, it is assumed that there is no genetic assortative mating among the twin parents, that genetic influences affect outcomes additively, and that genes neither interact nor correlate with environmental conditions (for a discussion of the assumptions and their implications for heritability estimates for status-related outcomes, see Diewald et al. 2015). It is possible to relax or test the assumptions of the CTD. To address potential gene–environmental interactions, for instance, researchers have conducted subgroup analysis (e.g., by parents’ socioeconomic status). It is also possible to account for nonadditive genetic influences: If the similarity in an outcome among MZ twins is more than twice that of DZ twins, it suggests the presence of nonadditive genetic influences. In such cases, one can estimate models that differentiate between additive and nonadditive genetic factors (e.g., Plomin et al. 2008).

Nevertheless, twin studies have often been criticized because genetic and environmental influences are not directly measured and hence remain black boxes. Newer methods rooted in modern molecular genetics allow those concerns to be addressed. Having measured genotype data does not make twin and family studies obsolete, however. In current research, a combination of pedigree data (such as nuclear or extended families, or multiple siblings from the same family) and measured genotype data represents a particularly promising approach to tackle the complex, intertwined web of causal pathways linking environments and genetics to outcomes (Young et al. 2019).

With the sequencing of the whole human genome, a new wave of molecular genetics has emerged (for an introduction, see Mills et al. 2020). Sequencing methods identify genetic variation in base pairs located in parts of the human genome. The most common genetic variations at a base position are single nucleotide polymorphisms (SNPs). To identify SNPs and their correlation with individual outcomes, researchers commonly use GWAS. In general terms, GWAS represent an atheoretical, regression-based approach to detect statistically significant associations between SNPs and the outcome of interest by scanning the entire human genome. Hence, GWAS are brute-force statistical searches for such correlations and require very large samples, as the impact of a single SNP is usually tiny.

Among the largest GWAS are the studies of educational attainment (EA). The most recent GWAS, EA4, was conducted in 2021 (Okbay et al. 2022); EA4 is based on more than 3.3 million individuals and explains about 16% of the total variance of educational attainment. To illustrate the rapid progress made in that field, EA1 was conducted in 2013, based on about 128,000 individuals, with an explanatory power of about 2% (Rietveld et al. 2013).

For the evaluation of GWAS results, it is important to keep in mind that results are highly dependent on the quality of the samples that are included in the GWAS database (for greater detail, see Mills and Rahal 2020; Popejoy and Fullerton 2016; Mills et al. 2020; Fatumo et al. 2022). Representativeness is one concern because random sampling is not an inclusion criterion, mostly because GWAS require enormous sample sizes. They are also typically performed on samples that include WEIRD individuals, i.e., relatively highly educated individuals from Western, industrialized, rich democracies. Samples tend to underrepresent minorities, and there is documented selection into genetic studies (Mills and Rahal 2020; Mills et al. 2020), even selection based on genetic variants (Popejoy and Fullerton 2016; Fatumo et al. 2022). These factors can have very large influences on the GWAS results. In addition, GWAS typically only assess associations with common genetic variants (i.e., > 1% prevalence), and any genetic associations uncovered are typically very noisy. Accordingly, the impact of rare genetic variants and variants with tiny effects may remain undetected.

Thus, current GWAS most likely pick up only a fraction of the relevant genetic influences that matter across different environmental settings. That means that GWAS capture the influence of SNPs that have been influential on average across historical time, countries, and the respective environmental conditions for the individuals of the sample. That the genetic effects reflect environmental influences has often been discussed in light of population stratification. Population stratification describes systematic differences in allele frequencies across different ancestry groups, which can correlate with behaviors. The classical example is the “chopstick gene”: In a sample consisting of individuals of Asian and European descent, one would identify genetic variants correlated with eating with chopsticks. These genetic associations would really be an effect of different culturally shaped practices between the two subpopulations rather than effects of genetic variants on eating with chopsticks. Care must be taken in order to avoid overinterpreting genetic effects as causal in such cases. Recent GWAS based on data on family members can help to alleviate this (Howe et al. 2021).

Results from GWAS can be used for different research purposes—for instance, to examine the genetic architecture of diseases and/or changes in genetic effects according to environmental conditions. A very common application in the social sciences is the construction of PGIs, which are ordinary variables created from genetic data using results from GWAS. These variables summarize an individual’s genetic profile with respect to a set of GWAS summary statistics. In practice, a PGI is a weighted sum score of SNPs that are common in the population and reflect an individual’s (genetic) disposition toward the trait that was the focus of the GWAS. The PGIs predict individual outcomes. For example, individuals who score high on a PGI that was created on the basis of the GWAS results for educational attainment have, on average, higher educational degrees than their counterparts who score low on the education PGI (Lee et al. 2018).

Researchers have been very enthusiastic about PGIs because they represent a straightforward approach to account for genetic confounding and can be used as any other control variable in ordinary statistical models. They are relatively easy to compute, and they are readily available in some of the well-known large-scale (household) surveys such as the Health and Retirement Study (HRS; Sonnega et al. 2014), the National Longitudinal Study of Adolescent to Adult Health (Add Health; Harris et al. 2019), and the Wisconsin Longitudinal Study (WLS; Herd et al. 2014), mostly for health, cognitive, and educational outcomes.

Despite current enthusiasm about PGIs, the limitations that we have discussed for GWAS apply to downstream PGIs as well. The PGIs are created as summary measures of a genome using weights from a set of GWAS results, i.e., summary statistics. Any bias or error in the GWAS will thus be included in the PGI. Accordingly, any PGI reflects an average genetic effect of genetic variants that were included in a GWAS, and it is highly dependent on the quality of the GWAS data. In addition, one may not be able to detect associations between genetics and the outcome of interest due to too small sample sizes. At the same time, PGIs might also reflect mechanisms that do not causally link genetic variants to outcomes. Take the example as provided by Pingault et al. (2022). Researchers may find a link between the PGI for depression and childhood trauma, indicating that individuals with a higher genetic risk for depression are more likely to have experienced such trauma. Yet this association could also be due to a common environmental cause, as individuals growing up in disadvantaged environments may be more likely to experience trauma and also have a higher genetic risk for depression. In light of different sources of bias that could drive a genotype–phenotype association, it is important to replicate studies using PGIs with other genetically sensitive designs and methods. Particularly, genotyped data in combination with family-based designs should be used to evaluate findings carefully in light of these sources of errors.

## Findings on Social Background Influences from Empirical Studies That Use the Education PGI

After the first GWAS for educational attainment (Rietveld et al. 2013), it was possible to create a PGI for educational attainment in other samples. The study of Conley et al. (2015) was one of the first to study social background effects while accounting for a PGI for education. Specifically, they studied how children’s educational attainment was affected by parental education and the child’s PGI for education. The inclusion of a child’s PGI partially captures direct genetic transmission, while this PGI for educational attainment (Rietveld et al. 2013) had rather limited explanatory power. Only about 15% of the zero-order correlation could be explained by it. This shows that the degree of genetic confounding is quite limited. Relatedly, the follow-up study by Liu (2018), which used a PGI with more explanatory power because it was based on a newer GWAS for education (EA3; Lee et al. 2018), still found only a relatively small degree of confounding. Similarly, Guo et al. (2022) analyzed the same PGI for education (EA3; Lee et al. 2018) as well as a PGI for cognitive abilities (using the 2018 GWAS of cognitive ability) to reassess the influence of socioeconomic status on the verbal ability of adolescents. They found that incorporating PGIs alongside traditional socioeconomic status measures significantly reduced the estimated effects of socioeconomic status (by about 10%–15%), indicating that a certain fraction of the impact previously attributed to socioeconomic factors was mediated by genetic predispositions. Whereas all of the aforementioned studies refer to the United States, recent findings for Norway support this tendency (Isungset et al. 2022). This study did not look at educational attainment but instead at test scores measured in childhood. The results show that parental education still has an association with a child’s test scores once a child’s PGI for education is controlled. Thus, even in Norway—a country context characterized by low levels of social inequality and comparatively high levels of equality of opportunity—evidence shows that the impact of social background persists and that the degree of genetic confounding is relatively small.

## Genetic Nurture: Its Emergence and What It May Mean

In 2018, two independent teams of geneticists reported a finding quickly denoted “genetic nurture” (Kong et al. 2018; Bates et al. 2018). Leading geneticists Augustine Kong and Tim Bates and their teams had used genotyped trio data—genotypes of the mother, the father, and their child—to estimate associations between parents’ genes and offspring outcomes (including educational attainment). This design enabled them to scrutinize both the impact of genes that parents biologically transmit and also the impact of genes that they *do not* biologically transmit. They used a so-called virtual parent design in which parents’ genetics that were *not *transmitted to the child were summarized in a separate PGI, which was then included in models. Any association between parents’ genes and children’s outcomes, net of the child’s own genes (the direct genetic effect), must operate through the environment. Such indirect genetic effects are environmentally mediated effects of parents’ genes. In effect, these studies used genetic data to estimate the impact of environmental/social transmission mechanisms. Indirect genetic effects or “genetic nurture” represent all causal effects flowing from parents’ own genetics through various mediators, such as parents’ skills and resources, to children’s outcomes. If indirect genetic effects operating through children’s environments are not accounted for in the analysis of direct genetic effects, these indirect genetic effects might be mistakenly treated as direct genetic effects. This can lead to an overestimation of the latter because children’s rearing environments are influenced by parental genotypes and are confounded with the children’s own genetic effects.

**Fig. 1:**
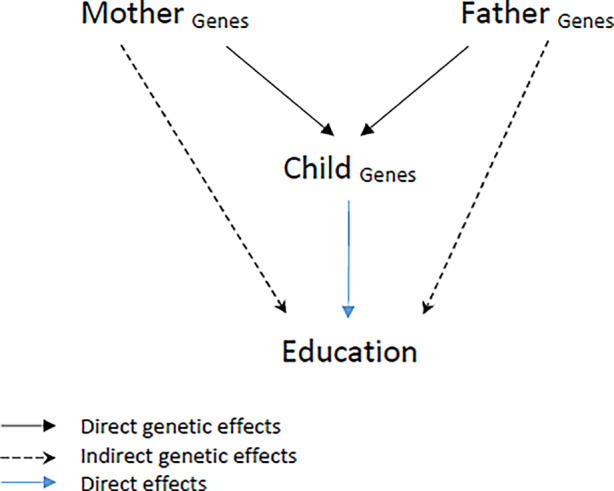
Indirect and direct genetic effects on children’s outcomes

The notion of genetic nurture represents an alternative way of conceptualizing (and estimating) genetic effects of parents on children’s outcomes. As can be seen in Fig. 1, parents’ genes can affect their children’s education directly through genetic transmission (black solid line). Genetics in turn has a direct effect on the outcome (blue solid line). Importantly, genetics affect children’s outcomes not only via biological transmission but also indirectly (black dashed line). Parents’ genetics shape parents’ behaviors (Avinun and Knafo 2014), which in turn influence children’s educational outcomes.

One type of mechanism that would lead to genetic nurture effects (i.e., the indirect genetic effects on children’s educational outcomes) that is often discussed in the literature invokes parental behaviors or investments. Specifically, indirect genetic effects may manifest in parents’ actions (parenting and/or investments), which then influence children’s educational outcomes. For instance, genetic influences that account for parents’ education or social status may enable them to spend more time with their children and to support their learning, which ultimately leads to more favorable educational outcomes.

The introduction of “genetic nurture” has inspired a lot of studies that investigated the presence or absence of such genetic nurture effects. A recent meta-analysis based on 12 empirical studies, mostly from the United States and the United Kingdom, concluded that genetic nurture accounts for about 8% of the variance in educational outcomes (including educational achievement and attainment), while direct genetic effects explain about twice as much (Wang et al. 2021). An even newer study in Norway (Isungset et al. 2021) confirmed this finding by looking at educational achievement; the impact of genetic nurture was about the same as that reported in the meta-analysis (Wang et al. 2021). Together, previous studies found that the impact of indirect genetic effects via genetic nurture and associated parenting behavior is smaller than initially assumed.

## Alternative Explanations for Genetic Nurture

Since the “discovery” of indirect genetic effects and the publications of the Bates and Kong teams (Kong et al. 2018; Bates et al. 2018), there has been a discussion on what genetic nurture means in substantive terms and whether these effects are robust to various methodological challenges. A main question has been whether the associations found between parents’ genetics and children’s outcomes, beyond the child’s own genetics, are truly the result of “nurture”—a purely environmental effect—or due to other types of mechanisms. For a “nurture” interpretation to hold, a causal interpretation of the indirect genetic effect is required. However, there are several threats to such causal claims. The literature discusses predominantly “dynastic effects” as alternative mechanisms underlying the indirect genetic effects (Morris et al. 2020; Kong et al. 2018; Bates et al. 2018; Koellinger and Harden 2018; Mills et al. 2020; Novembre et al. 2008).

Dynastic effects emerge because parents transmit genes and the rearing environment simultaneously. For instance, parents with many genetic variants found to be linked with education (or, in more practical terms, a high PGI for education) not only transmit genetic influences that are linked to higher education, but they also often provide a more intellectually stimulating home environment, have more financial resources, or systematically differ from other parents in ways that ultimately affect children’s outcomes. Because parents transmit both genetics and the rearing environment, any association between a measure of parents’ genetics, such as a PGI, and children’s outcomes may also reflect such effects.

Dynastic effects may also arise from mating patterns. Assortative mating with respect to education, for instance, is well established in Western societies (Kalmijn 1998). A strong similarity in educational attainment is likely accompanied by stronger similarity in education-relevant genetics. Such genetic assortative mating complicates genetic analysis and the interpretation of its findings. In twin studies, for example, it can lead to an underestimation of heritability estimates and an overestimation of shared environmental influences. In studies on the impact of PGIs, genetic assortative mating may induce bias that substantively goes in the other direction: It can inflate the observed association between parents’ PGIs and their children’s educational outcomes. Because there is likely strong assortative mating on alleles relevant to educational attainment, due to the widespread phenotypic educational homogamy (Mare 2016; Birkelund and Heldal 2003), this may also explain the presence of indirect effects of parents’ genetics on children’s outcomes.

In light of such “dynastic effects,” more rigorous research designs are required to validate indirect genetic effects as causally flowing from parents’ own genetics and related behaviors to children’s outcomes, i.e., the nurture-type mechanism. One very recent example of a stronger empirical test is the study from Nivard et al. (2024), in which the authors were able to explicitly test whether nurture or dynastic effects were behind genetic nurture-type associations.

Nivard et al. (2024) used the Norwegian Mother, Father and Child Cohort study (MoBa), which is a very large observational pregnancy study (Magnus et al. 2006, 2016). The outcome was educational achievement, measured by scores on standardized national tests in math and reading administered in the fifth to ninth school grades. National tests are taken at school and are objectively scored.

The MoBa study offers data on genetic information on trios (i.e., mother, father, and their child) and also has a link to the Norwegian population registers. These registers include links between parents and children. After selecting complete trios, i.e., family units in which the mother, father, and child were all genotyped, Nivard et al. (2024) identified parents who had a sibling who also participated in the MoBa study. This extended pedigree design—including children, their parents, and the siblings of the parents—represents a unique opportunity to separate dynastic effects from genetic nurture effects.

The inclusion of parents’ siblings is what enabled the researchers to decompose the indirect genetic effect identified by Kong et al. (2018) and Bates et al. (2018) into two components: one that operates between families and one that operates within families. Specifically, Nivard et al. (2024) computed a within-sibship mean of the PGIs of a parent and his or her sibling (the between-family component) and a deviation score (the within-family component) for that parent. The between-family component represents the part of indirect genetic effect that is generated by dynastic effects, i.e., extended families being different from each other. The deviation of the parent from the sibship average captures the within-family indirect genetic effect that represents the genetic nurture mechanism. The estimation strategy resembles a sibling fixed-effects design with deviation and mean scores in the parental generation, and it allows estimation of both the between-family and the within-family factors. Because Nivard et al. (2024) included multiple outcome measures, a random effect for each test is also included, and the model corresponds to a simple multilevel model (Goldstein 2011). The study was shown to have sufficient statistical power to detect nurture-type effects comparable in size to what was reported in the meta-analysis conducted by Wang et al. (2021). Nivard et al. (2024) used three different PGIs calculated from the standard educational attainment GWAS, a combined GWAS for cognitive and noncognitive skills, and a within-family GWAS less prone to confounding.

The separation of the two components demonstrated that the within-family component, representing the genetic nurture component, was indeed rather small and not statistically significant. Results showed that indirect genetic effects of parents’ education-related PGIs on children’s academic achievement cannot be solely accounted for by processes that occur exclusively within the nuclear family.

Rather than reflecting effects flowing from the parents’ genotype through environmental mechanisms to child achievement, indirect genetic effects seem to reflect consequences of dynastic processes. The between-family parameter in the model represents the association with the average PGI of the siblings, which basically is a proxy for the child’s grandparents’ genetics. This parameter measures to what extent broader family genetics are associated with children’s outcomes. A multigenerational process of social stratification as well as assortative mating among parents are key mechanisms that can account for such a gene–environment correlation.

While Nivard et al. (2024) find that dynastic effects play a much more important role than previous studies suggested, their evidence does not allow us to conclude that genetic nurture effects do not exist at all. If genetic nurture exists, however, its importance for the variation in children’s educational achievement seems to be rather small. Relatedly, the study focused on the genetic effects from parental PGIs linked to educational achievement. Thus, any parenting behavior that is unrelated to education PGIs is not captured by the study design. There may be other, important aspects of parents’ psychology and behavior that are unrelated to the genetics of education but still affect children’s academic performance. However, the correlation between education-related genes and genes correlated with personality, risk aversion, and cognitive and noncognitive skills is quite high (Demange et al. 2021). Nevertheless, we cannot conclude that parenting does not affect children’s schooling outcomes. Yet with respect to indirect genetic effects on their offspring’s educational achievement, parents seem to play a smaller role than earlier studies suggest. It should also be noted that the heritability reported for educational achievement typically is higher than what is reported for adult educational attainment.

In conclusion, initial studies by Bates et al. (2018) and Kong et al. (2018) highlighted substantial genetic nurture effects. These studies could be interpreted as evidence for mechanisms typically cited in sociological theory linking parents’ socioeconomic status to children’s outcomes. However, these studies did not fully account for dynastic effects, which are possible drivers of indirect genetic influences on educational outcomes. The study by Nivard et al. (2024) was among the first to apply a more sophisticated research design by incorporating siblings of parents in addition to trios of mothers, fathers, and children. This design made it possible to differentiate between within-family “genetic nurture effects” and the broader “dynastic effects.” Their findings showed that the impact of genetic nurture was less substantive than previously believed, particularly when compared to dynastic effects. This finding underscores the importance of the broader multigenerational processes that extend beyond the nuclear family as drivers of indirect genetic effects. The Nivard et al. (2024) study is an early example that demonstrates how a combination of a family design and direct measures for genetics can help to elucidate what lies behind indirect genetic effects. This finding indicates that many plausible mechanisms proposed in the stratification literature that link parents’ skills, knowledge, and resources to children’s outcomes remain incomplete. The Nivard et al. (2024) results show that the social mechanisms that matter for educational achievement reflect to a large extent dynastic effects and operate across generations and, hence, outside the nuclear family. Such multigenerational stratification processes may include the transmission of wealth, the broader family network or climate, the neighborhood, and mating choices, all of which currently play a subordinate role in current theories on the intergenerational transmission of educational inequality.

More recently, several papers have emerged that shed further light on the processes driving indirect genetic effects. It is clear that assortative mating has not gotten the attention it deserves, neither substantively nor methodologically. As noted above, assortative mating may bias heritability coefficients from twin studies downward. Previous twin-based analyses have shown a significant reduction in heritability estimates for educational attainment once assortment was accounted for (Baier and Lang 2019; Baier et al. 2022; Wolfram and Morris 2023). Evidence based on molecular studies supports the role of assortative mating. For instance, a recent study using the Norwegian MoBa data confirmed the presence of assortative mating across several generations and revealed that genetic similarities within families are indeed markedly higher than one would expect under the assumption of random mating (Torvik et al. 2022). Similarly, a study on the inheritance of social status in England using broad historical data sources reported that once assortative mating was taken into account, a simple genetic model would explain parent–offspring associations quite well, without any sociocultural transmission operating at all (Clark 2023). Acknowledging the presence of assortative mating, a recent methodological study provided tools for estimating PGIs in which the potential bias from assortative mating was removed (Young 2023). In light of these findings and such methodological advances, in the near future we will most likely learn even more about the nature of indirect effects and what lies behind them.

## What Are Current Results Pointing Toward?

The purpose of this paper was to highlight the relevance of current trends in sociogenomics research for social stratification theory, particularly educational inequality. When we summarize what we can learn about social background effects on children’s educational outcomes from findings based on molecular genetics today, there are at least three important takeaways.

First, it is clear that the child’s own genetics are an important predictor of educational outcomes. This concurs with the literature based on twin and family studies, which assigns a high proportion of the variance in education to genetics and relatively small proportions of the variance to shared family environments (Branigan et al. 2013; Polderman et al. 2015). Thus, genetic transmission mechanisms represent an essential pathway through which educational attainment is transmitted from one generation to the next. This finding already requires a shift in standard sociological perspectives on the intergenerational transmission of educational inequality. Most approaches focus mainly on social transmission mechanisms while not emphasizing the genetics of the child. Conventional stratification theory sometimes acknowledges genetic influences as a determinant of educational achievement (i.e., what educational sociologists have denoted primary effects), but it has not incorporated the role of genetics for later educational stages and posteducation outcomes such as occupational prestige or social class. For instance, in the recent update of Goldthorpe and colleagues’ influential theory on the intergenerational reproduction of class positions (Bukodi and Goldthorpe 2022), genetics was not mentioned despite the evidence of nontrivial genetic components in many outcomes related to social stratification and class (Nielsen and Roos 2015; Nielsen 2006; Van Hootegem et al. 2024).

Second, once education PGIs are accounted for, the estimates of social background associations still remain. In other words, the impact of parental social background holds even if we account for direct genetic transmission. Merely including a measure of the child’s own genetics does not “control away” associations between social background and child outcomes. There are residual associations with parental education even when the parents’ and child’s PGIs are controlled (Liu 2018; Isungset et al. 2022). However, it is important to note that PGIs can only provide partial control for genetics (Zietsch et al. 2023). Not only are scores limited to the specific phenotypes for which a solid set of GWAS results is available, but PGIs are also very noisy predictors. The residual associations of parents’ status and child outcomes may decrease once better and more precise PGIs are available. However, these associations could also represent actual causal effects of parents’ education on children’s outcomes.

Third, indirect genetic effects are an interesting and relevant mechanism driving educational inequality. Indirect effects in the spirit of “genetic nurture” have received a lot of attention in the literature and fit standard sociological narratives on the roots of educational inequality: Education-minded parents’ nurturing behaviors and investments lead to their children doing better in school and having better outcomes in the educational system (Wang et al. 2021). However, indirect genetic effects can also be driven by dynastic inheritance of relevant resources. The Nivard et al. (2024) study differentiated between these different types of indirect genetic effects and demonstrated that the size of the nurturing effect has previously been overstated—at least for the Norwegian context. Accordingly, the larger share of indirect genetic effects is dynastic in nature and may be generated by some kind of multigenerational social stratification process or genetic assortative mating (i.e., that parents are genetically similar).

What can we conclude about social background effects on children’s educational outcomes based on the set of recent findings using molecular genetic data and methods?

The findings call for revisions of conventional sociological perspectives on the mechanisms that contribute to educational inequality. Sociological stratification theory places quite a bit of emphasis on active parenting and nurturing, but the processes that drive the intergenerational association with children’s educational outcomes seem to be more complicated than what is offered by current theory. Including modern genetics in this interdisciplinary research endeavor can advance theory development, as the latest findings suggest that social background effects are at least partly dynastic and a result of subtle gene–environment correlations rather than a result of mechanisms operating within the nuclear family context. Substantively, this means that social background effects on children’s educational outcomes are less a result of parents’ own genetically anchored parenting styles, investments, skills, talents, and abilities than one might have thought. Instead, the new evidence on indirect genetic effects means that either socially transmitted resources from the wider family network affect children’s educational achievements or indirect effects reflect parents’ genetic similarity induced by assortative mating. Importantly, to fully understand the dynamics underlying indirect genetic effects, theoretical insights from sociologists and related disciplines are essential. Their expertise is crucial in understanding how these genetic influences interact with broader environmental factors and social structures and also how selective mating choices come about.

Let’s consider the scenario of a dynastic world and leave the potential role of assortative mating aside for a while. How can we theorize a dynastic world in which children’s educational achievements are influenced by social advantages “inherited” from their parents or extended family, while these advantages remain unrelated to their parents’ own genetic dispositions for education? These influences could stem from peers or comprise other network effects that emerge due to residential segregation or school effects. While these influences seem reasonable, evidence—particularly from Norway, where some of the reviewed results were obtained—suggests that such effects are not meaningful (Hermansen et al. 2020). Since we cannot pinpoint the exact mechanism that is driving dynastic effects, we can only speculate. Some candidates are the transmission of wealth across generations or other features of family-learning cultures. For instance, extended family members, such as grandparents, who engage in reading activities or other intellectually stimulating activities with their grandchildren may contribute to improved test scores in children. Yet such explanations must nevertheless also fit established findings in multigenerational stratification research. If indirect genetic effects really reflect consequences of grandparents’ status, then why are effects of grandparental status on children’s outcomes found to be miniscule (e.g., Engzell et al. 2020)? Perhaps one reason why it is challenging to conceptually grasp the underlying mechanisms is that they work so differently from how we have been taught and how we are used to thinking about social background effects.

We are left with thought-provoking results about how social background effects work and, more importantly, how they do not seem to work. Social science research using molecular genetic data is a rapidly evolving field, and the next few years will provide us with more possibilities to test alternative explanations in a more robust way.

First, more data sources will be publicly available as researchers from various disciplines increasingly acknowledge that their explanations remain incomplete if genes are ignored. Thus, sample sizes for GWAS are increasing, which in turn increases the accuracy of PGIs. Currently, the majority of participants whose data are used in GWAS have European ancestry. This limits our knowledge about genetics and genetic variations across social contexts to a limited set of human populations. It is hoped that samples will not only increase in size but will also become more diverse in terms of ancestries and representation of different regions of the world.

Second, there will be more opportunities and efforts to link different data sources. For instance, emerging genetically informed observational studies can be linked to register data. This offers new possibilities to exploit family designs, as family members of survey participants are identifiable in the registers. Increasing availability of within-family genomic data, in which multiple family members are genotyped, will improve possibilities for identifying genetic effects with fewer of the biases discussed above. Such developments have led to methodological advances such as within-family GWAS to improve estimations of direct genetic effects (for education, see Okbay et al. 2022).

Taken together, the recent methodological developments and related findings in this literature deserve greater attention from stratification scholars, who for a long time have not considered genetics in their models of intergenerational transmission. A sound understanding of indirect genetic effects represents an important next step for future research and is an interdisciplinary research endeavor that clearly advances our understanding of how educational inequality is reproduced across generations.
